# IFN-α Is Constitutively Expressed in the Human Thymus, but Not in Peripheral Lymphoid Organs

**DOI:** 10.1371/journal.pone.0024252

**Published:** 2011-08-31

**Authors:** Arnaud D. Colantonio, Marta Epeldegui, Maria Jesiak, Loes Jachimowski, Bianca Blom, Christel H. Uittenbogaart

**Affiliations:** 1 Department of Microbiology, Immunology, and Molecular Genetics, David E. Geffen School of Medicine, University of California Los Angeles, Los Angeles, California, United States of America; 2 Department of Pediatrics, David E. Geffen School of Medicine, University of California Los Angeles, Los Angeles, California, United States of America; 3 UCLA AIDS Institute, David E. Geffen School of Medicine, University of California Los Angeles, Los Angeles, California, United States of America; 4 Jonsson Comprehensive Cancer Center, David E. Geffen School of Medicine, University of California Los Angeles, Los Angeles, California, United States of America; 5 Academic Medical Center, University of Amsterdam, Amsterdam, The Netherlands; New York University, United States of America

## Abstract

Type I interferons have been typically studied for their effects in the context of bacterial or viral infections. However in this report, we provide evidence that Interferon-alpha (IFN-α) expressing cells are present in the thymus in the absence of infection. We show that pDC express the highest level of IFN-α and that MxA, which is exclusively expressed after engagement of the type I IFN receptor by IFN-α/β, is expressed in normal fetal and post-natal thymus, but not in the periphery. The highest level of MxA is expressed in mature thymocytes and pDC located in the medulla and at the cortico-medullary junction. The anti-microbial peptide LL-37, which is expressed in the thymus, when complexed with eukaryotic nucleic acids, induces the secretion of IFN-α by thymic pDC. This results in the upregulation of MxA expression in responsive thymocytes. We propose that the secretion of IFN-α in the thymus may function to regulate the rate of T cell development and modulate the requirements for the selection of developing T cells.

## Introduction

Type I interferons (IFN) are immunomodulatory cytokines that function to alert cells to the presence of pathogens [1]. Antiviral activity of type I interferons is mediated by the expression of interferon stimulated genes (ISG), which is dependent on signaling through the IFN-α receptor (reviewed in[2]). IFN-α receptor signaling leads to phosphorylation of STAT1/2 and results in the expression of interferon regulatory factor 7 (IRF-7), required for the transcription of downstream ISG. Upregulation of ISG prevents the spread of viral infection through several mechanisms including the specific degradation of viral gene products, inhibition of protein translation, and ultimately apoptotic cell death. One ISG, Myxovirus resistance A (MxA) has been linked with resistance to viral infection [3], [4], [5]. MxA protein inhibits the viral life cycle at three distinct steps, including nucleocapsid transport to the nucleus [6], transcription of viral gene products [7], or viral assembly [8]. Expression of this particular ISG is tightly regulated and only expressed when IFN-α is secreted [9]. Expression of MxA has been widely utilized as a bio-marker for secreted IFN-α/β in both viral and bacterial infections [10], [11], [12].

In addition to antiviral effects, type I interferons are known to have immunoregulatory activities, such as suppression of murine T and B cell development *in vivo* [13], [14], [15]. We previously showed that IFN-α suppresses the development of human T cells *in vitro* by inhibiting early steps of T cell development [16]. Thus, in addition to its antiviral effects, IFN-α may play a regulatory role in the thymus. We previously identified IFN-α positive cells in normal thymus tissue in the SCID-hu mouse model [17]. However, both the nature of the IFN-α expressing cells and the stimulus that induced IFN-α remained elusive. The purpose of the current study is to further characterize IFN-α expressing cells in normal thymus tissue, compare these cells to those found in peripheral lymphoid tissues, and to examine the trigger for IFN-α production in the absence of infection.

Although every white blood cell has the ability to produce IFN-α, plasmacytoid dendritic cells (pDC) are the highest producers of type I IFN. In the thymus pDC are located in the thymus medulla [18], [19] and play a role in the induction of regulatory T cells [20], [21]. The primary function of peripheral blood and peripheral lymphoid pDC is to secrete large amounts of IFN-α/β in response to viral and bacterial infection [18], [22], [23], [24], [25], [26], [27], [28]. pDC sense infection via the expression of the Toll like receptors (TLR) -7 and -9, which bind single stranded RNA and hypomethylated CpG DNA motifs, respectively. While thymic pDC show some immunophenotypic differences compared to peripheral pDC [19], we have shown they retain the ability to secrete IFN-α in response to CpG [17].

Peripheral blood pDC may also secrete IFN-α in response to tissue damage as observed in the skin lesions in psoriasis [29]. In this case, the TLR-7/9 stimulus is eukaryotic nucleic acids, RNA or DNA, in complex with an anti-microbial peptide (LL-37) derived from the CAMP gene [29], [30]. LL-37 mRNA is expressed in the thymus [31], but it has not been investigated whether the resultant peptide can stimulate thymic pDC in the presence of autologous DNA or RNA. Given that thymocytes undergoing negative selection die through induction of apoptosis [32] it is attractive to speculate that this process provides an ample source of self-RNA/DNA, which in complex with LL-37 induces the secretion of IFN-α from pDC.

In this report we not only examine IFN-α expression in thymocytes, but also expression of surrogate markers that are indicative of type I interferon receptor signaling activity, including MxA, IRF-7, and phosphorylated STAT1 (pSTAT1). We report that IFN-α and MxA are constitutively expressed in normal postnatal and fetal thymus tissues *ex vivo,* corroborating our previous results that IFN-α and MxA are expressed in normal thymus/liver implants in the SCID-hu mouse [17]. Furthermore, MxA, IRF-7, and pSTAT1 proteins are preferentially expressed in mature thymocytes that are located in the thymic medulla. Not only does this area coincide with the known localization pattern of pDC, but in addition we observed that pDC constitutively expressed the highest levels of IFN-α in the normal thymus. Furthermore low, but reproducible, expression of LL-37 was detected within the thymic medulla. This, together with our observation that LL-37 complexed with eukaryotic RNA or DNA can stimulate pDC to express IFN-α, suggests that thymic pDCs are involved in LL-37/DNA-RNA induced secretion of IFN-α in the absence of infection. We speculate that thymic pDC may play a role in regulating the rate of normal T cell development or alter the requirements for negative selection.

## Materials and Methods

### Ethics statement

The use of anonymous thymus, peripheral lymphoid and fetal liver tissues and peripheral blood mononuclear cells was reviewed by the UCLA Institutional Review Board (IRB), which concluded that these activities did not involve human subjects, and therefore did not require IRB review or certification.

### Reagents and mAbs

mAbs CD1a, CD3, CD4, CD8, CD27, CD45RA, CD123, and STAT1 (pY701); isotype controls IgG1 and IgG2 conjugated with FITC, PE, PerCP, PE-Cy7 or APC; and goat anti-mouse FITC were obtained from Becton Dickinson (BD); CD1a conjugated with PE from Beckman Coulter Immunotech; BDCA4-APC from Miltenyi Biotec; CD8-APC-Alexa750, CD45RA-PerCP-Cy5.5, CD1a-Pacific Blue and CD3-650 eFluor (Nano-crystals) from eBioscience; IFN-α -biotin (Endogen); isotype control IgG2a pure, Goat anti-Rabbit PE from Caltag; mAb to CD27 from Lab Vision; Goat anti-mouse IgG1 AlexaFluor-488, Goat anti-mouse IgG2a AlexaFluor-594, Goat anti-Rabbit AlexaFluor-594, and Zenon anti IgG2a AlexaFluor-488 from Molecular Probes. mAb to MxA was generously provided by Drs. Haller and Kochs (University of Freiburg, Germany). mAb CD1a pure, TBST, Antibody Diluent, and Target Retrieval Solution were obtained from DAKO (Carpinteria, CA); rabbit anti-mouse IgG1 pure from Zymed; mAb IRF-7 from Santa Cruz Biotechnology; mAb LL-37 from Cell Sciences. Mounting Medium for fluorescence was obtained from Vector Laboratories. Polyoxyethylenesorbitanmonolaurate (Tween-20) was purchased from Sigma-Aldrich, paraformaldehyde from Polysciences. Recombinant human IFN-α was obtained from BioSource.

### Cell cultures

Fetal tissues were obtained from Advanced Biosciences Resources; adult PBMC from the UCLA CFAR Virology Core Laboratory; normal human postnatal thymus specimens from children undergoing corrective cardiac surgery. Thymocytes were prepared and cultured, as previously described, in serum-free medium consisting of IMDM (Omega Scientific) supplemented with delipidated BSA (Sigma-Aldrich) at 1100 µg/ml, transferrin (Sigma-Aldrich) at 85 µg/ml, 2 mM glutamine, and penicillin/streptomycin at 25 U/25 µg/ml[17]. Thymocytes were cultured at 1–2×10^7^ cells/ml in serum-free medium as pellet cultures at 37°C in 5% CO_2_ in round-bottom tissue culture tubes.

### Immunofluorescent intracellular staining and flow cytometry

For combined surface and intracellular staining using the unconjugated mAb to MxA, 1×10^6^ cells were fixed in 1% paraformaldehyde, permeabilized with 0.2% Tween-20, and stained intracellularly with a 1/500 dilution of mAb to MxA or isotype control Ab for 20 min at 4°C as previously described [17]. The cells were washed with 0.2% Tween-20, and goat anti-mouse FITC mAb was added at 10 µl/tube and incubated for 20 min at 4°C. Cells were washed with 0.2% Tween-20 and incubated for 10 min with 50 µl/tube of a 1/15 dilution of normal mouse IgG (Caltag Laboratories). PE, PerCP, and APC-conjugated mAb were added for 20 min at 4°C and washed with 0.2% Tween-20. Cells were finally resuspended in FACS buffer before acquisition on a FACSCalibur flow cytometer (BD Immunocytometry Systems).

For combined surface and intracellular staining using unconjugated IRF-7, 1×10^6^ cells were first surface-immunophenotyped with FITC, TC, or APC antibodies and fixed with 1% paraformaldehyde. Cells were permeabilized with 0.2% Tween-20 and incubated with 400 ng IRF-7 antibody for 40 minutes. Cells were washed and incubated with 10 µl Goat anti-Rabbit PE for 20 minutes. Cells were resuspended in FACS buffer before acquisition on a FACSCalibur or LSRII flow cytometer.

For combined surface and intracellular staining using IFN-α-biotin, 1×10^6^ cells were first surface-immunophenotyped with FITC, PerCP-Cy5.5, PE-Cy7, APC, APC-Ax750, Pacific Blue and eFluor650 nanocristal antibodies, then fixed with BD FACS Lysing Solution. Cells were then permeabilized with BD FACS Permeabilizing Solution 2 and incubated with 2 µg of IFN-α-biotin (Thermo Scientific) or mouse IgG1 (BD Biosciences) antibody for 20 minutes. Cells were then washed and incubated with 5 µl of SAV-PE (BD Biosciences) for 10 minutes. Cells then were washed and resuspended in FACS buffer before acquisition on a LSRII (BD).

For combined surface and intracellular staining using PE conjugated pSTAT1, 1×10^6^ cells were washed with FACS buffer and surface-immunophenotyped with FITC and APC labeled antibodies. The cells were fixed in 1.5% paraformaldehyde for 10 minutes at room temperature and permeabilized with 1 ml cold (4°C) methanol for 10 minutes at 4°C. After washing the cells twice with FACS buffer, they were stained with 10 µl pSTAT1-PE or IgG2-PE control antibody for 30 minutes at room temperature. The cells were resuspended in 250 µl FACS buffer before acquisition on a FACSCalibur or LSRII flow cytometer. Multiparameter data acquisition was performed with CellQuest software (BD). Multiparameter data analysis was performed with FCS Express Version 3 (Denovo Software).

### Immunofluorescence microscopy

Fetal tissues and post-natal thymus were fixed in formalin and embedded in paraffin by the UCLA Tissue Procurement Core Laboratory. Four µm sections were cut and placed on slides. The slides were heated at 60°C for 30 minutes and washed in xylenes, followed by rehydration in 100%, 95%, and 70% ethanol and incubation in target retrieval solution pH 9.0 for 20 minutes at 95–99°C. Samples were stained with monoclonal antibodies to MxA (1∶500), IRF-7 (1∶50), or LL-37 (1∶50) and CD1a (1∶50) or CD27 (neat) overnight at 4°C. Samples were washed and stained with secondary antibodies (1∶250) for 4 hours at room temperature. Slides were mounted in fluorescence mounting medium and coverslips were sealed with clear nail polish. For staining with CD123 and MxA slides were stained first with CD123 (1∶40) overnight and washed. Slides were then stained with rabbit anti-mouse IgG1 (1∶500) for 2 hours followed by Goat anti Rabbit AlexaFluor-594 for 2 hours. MxA was conjugated with Zenon anti IgG2a AlexaFluor-488 following the manufacturer's protocol. Slides were stained for 2 hours and fixed with 2% paraformaldehyde for 10 minutes before samples were mounted in fluorescence mounting medium and coverslips were sealed with nail polish. Images were taken with a Zeiss compound microsope and analyzed with Axiovision LE version 4.6 (Carl Zeiss Microimaging).

### Immunohistochemistry

Postnatal thymus and fetal tissues were formalin fixed and paraffin embedded. 4 um sections were mounted on slides and stained with a monoclonal antibody to LL-37 (1∶50) or CD19 (1∶50) and control IgG2a antibody followed by biotinylated rabbit anti-mouse antibody and streptavidin-biotin-peroxidase complex using the DAKO Catalyzed Signal Amplification System.

### Isolation and stimulation of thymic pDC

pDC were isolated from human thymus tissue by MACS-based negative selection (Miltenyi). The purity of pDC was checked by flow cytometry using CD45RA FITC, BDCA4-APC, CD123PE, CD3 PerCP antibodies. Cells were stained with 7AAD to determine the percentage of apoptotic cells.

pDC were stimulated with CpG-A(2216) oligonucleotides (6 µg/ml) or LL-37 (10 µg) in the presence or absence of DNAse I (Sigma; final conc.5 µg/ml). LL37 premixed with 2 µg autologous DNA (DNAsy Kit, Qiagen) (peptide: DNA mass ratio of 5∶1) and incubated 30 min. at room temperature (50 µg/ml of LL-37 and 10 µg/ml of DNA final concentration) was added to pDC in some experiments. After overnight culture (18–20 hours) at 37°C, 5%CO_2_ IFN-α level was measured by enzyme-linked immunosorbent assay (ELISA; PBL Biomedical Laboratories). Remaining cells were frozen in Trizol and used to isolate RNA for quantitative real-time PCR for MxA expression.

To obtain a purified population of pDC the cells were first enriched with BDCA4 selection kit (Miltenyi) followed by cell sorting of CD123^+^CD45RA^+^ cells using a FACSAria II cell sorter (Becton Dickinson). The purity of sorted pDC exceeded 95%.

pDC were stimulated with CpG-A(2216) oligonucleofotides (10 µg/ml) or LL-37 peptide (Netherlands Cancer Institute) (LLGDFFRKSKEKIGKEFKRIVQRIKDFLRNLVPRTES) (50 µg/ml) in the presence or absence of DNAse I (Roche) (2000 U/ml) and RNAse (Roche) (50 ug/ml).

LL-37 (50 µg/ml) was premixed with eukaryotic DNA (10 µg/ml) and RNA from cell line supernatant for 30 min. at room temperature and added to pDC in some experiments. After 4 hours of culture at 37°C, 5% CO_2_ pDC were resuspended in Trizol. RNA was isolated and cDNA synthesis was performed by using the transcription high fidelity cDNA synthesis kit (Roche).

### RNA isolation and real-time PCR

10×10^7^ cells were frozen in TriReagent (Invitrogen) and stored at −80°C until RNA was isolated, when samples were incubated with poly-acryl carrier for 5 minutes at room temperature, 100 ul 1-Bromo-3-chloropropane per mL of TriReagent was added for 10 minutes, spun at 12,000 g for 10 minutes at 4°C and the aqueous phase was transferred to a fresh tube. 500 uL of isopropanol was added for 10 minutes and samples were spun at 12,000 g for 8 minutes at room temperature. Pellets were washed with 75% ethanol and allowed to air dry before resuspension in 100 µl water.

Taq-man gene expression assays were used to measure gene expression (Applied Biosystems). Real-time PCR for analysis of MxA (Mx1 HS_00182073_m1) IFNA1 (Hs00256882_s1), IFNA2 (Hs00265051_s1) was performed following manufacturer's instructions. 96 well plates were run on an ABI 7300 and analyzed with the 7300 system software.

Q-PCR on sorted pDC and thymocytes for relative expression of IFN-α and MxΑ mRNA was performed by Sybr Green PCR (iCycler Bio-Rad CFX96). Primers: IFN-α: 5′-GACTCCATCTTGGCTGTGA-3′, 5′- TGATTTCTGCTCTGACAACCT-3′, MxA: 5′-GGTGGCTGAGAACAACCTGT-3′, 5′-GGTCCTGCTCCACACCTAGA-3′, Beta-Actin and GAPDH were used as controls. (Beta-Actin: 5′-CTCTTCCAGCCTTCCTTCCT-3′, 5′-AGCACTGTGTTGGCGTACAG-3′) (GAPDH: 5′- GAGTCAACGGATTTGGTCGT-3′, 5′-GACAAGCTTCCCGTTCTCAG-3′).

### Statistics

Non-parametric Spearman rank correlation was used to compare percent MxA expression versus age of thymus tissue; two sided Wilcoxon-Mann-Whitney test to compare mean total MxA expression between males and females; two sided Wilcoxon signed rank sum test and sign test to compare mean fluorescence intensity of MxA in thymocyte subsets.

## Results

### Expression of MxA in thymus, but not peripheral lymphoid organs

Previously we observed that IFN-α was detected in normal human fetal thymus/liver implants from SCID-hu mice [17]. The current study was designed to obtain further insight in the localization of endogenous constitutively produced IFN-α, the cell type and the mechanism of induction in the thymus. The inability to reliably detect all IFN-α subtypes and differences in the kinetics of IFN-α secretion by different cell types makes the study of IFN-α secretion technically challenging. However, studies have shown that the ISG MxA is only synthesized in the presence of type I IFNs, and is absent when type I IFNs are not secreted [9] and requires signaling though STAT1 [33]. Hence, we first set out to analyze the expression of MxA as a surrogate marker to detect the presence of secreted type I interferons.

**Figure 1:**
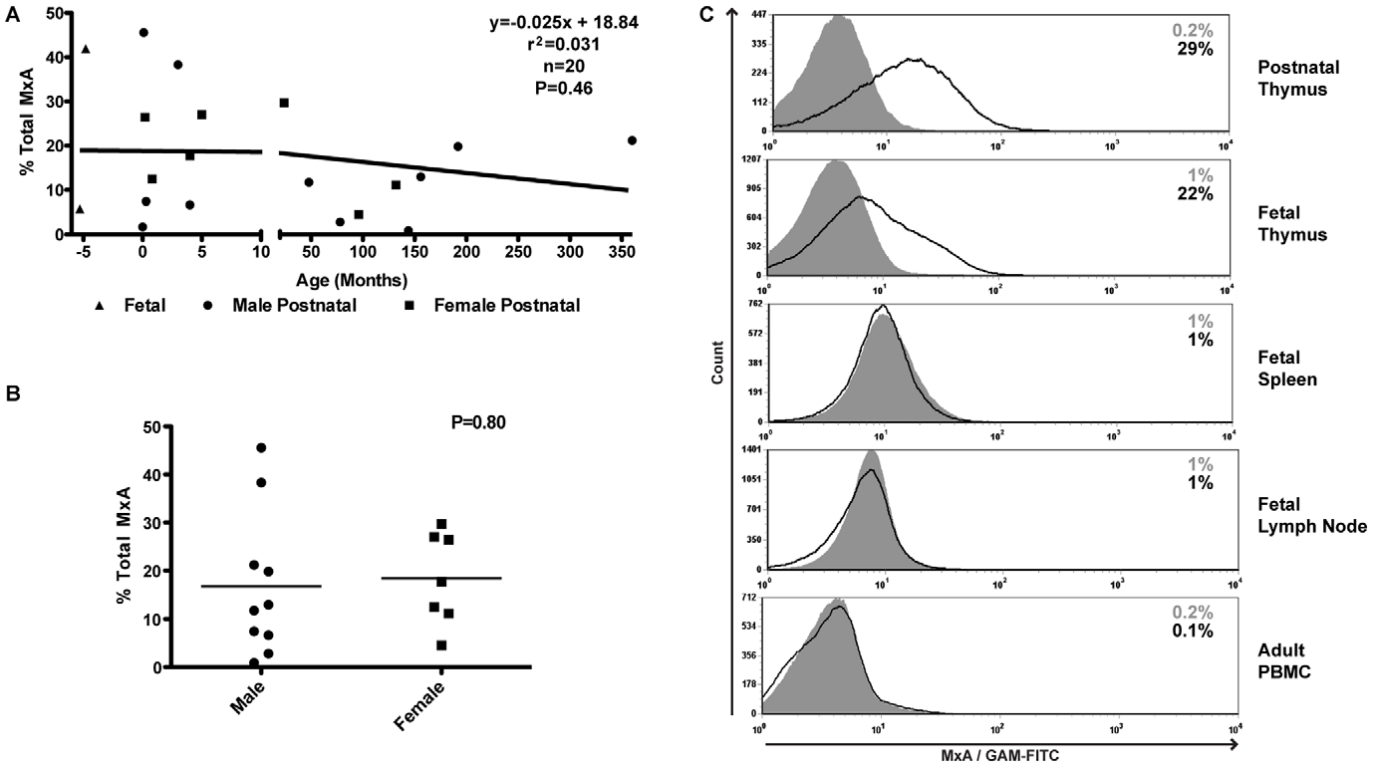
Constitutive expression of MxA in thymocytes. *A,B*, Total thymocytes were isolated from fetal and postnatal thymus tissues. Cells were stained for surface expression of CD3, CD4, CD8 and intracellular expression of MxA *ex vivo*. Three fetal and 18 postnatal thymus tissues were examined to determine the effect of age (A) and sex (B) on MxA expression. *C*, Cells from postnatal thymus, fetal thymus spleen and lymph node tissues and adult PBMC were stained for intracellular expression of MxA. Fetal tissues were obtained from the same donor.

We confirmed that type I IFNs are constitutively secreted in the thymus by analysis of intracellular MxA expression using flow cytometry (Fig.1). We extended these findings by showing that postnatal (18/18 donors, age 1 day-30 years) and fetal thymus (3/3 donors) expressed MxA (Fig.1A). While MxA was detected in all thymus tissues (range 0.9–45%), there was no correlation between total MxA expression and age (R^2^ = 0.0301, p = 0.46) (Fig. 1A) or mean total MxA expression and sex (p = 0.80) (Fig. 1B).

The presence of IFN-α secretion and resulting MxA expression in normal tissues may not be unique to the thymus. To investigate this, we compared the MxA expression in different lymphoid tissues by staining postnatal thymus and fetal thymus, spleen, and lymph node from the same fetal donor. In 3/3 experiments, only the thymus, but not spleen or lymph node from the same fetal donor expressed MxA (Fig. 1C). Also adult PBMC (n = 3) (Fig. 1C) lacked MxA expression. These findings were confirmed at the RNA level by real time PCR (data not shown). Thus, MxA expression in the thymus is not a result of localization of cells within lymphoid tissue as both fetal spleen and lymph nodes lack MxA expression. This finding is of note since fetal tissue by definition is devoid of bacterial or viral infections, yet the presence of MxA in the thymus suggests that IFN-α/β is secreted constitutively.

### MxA expression is located in the thymic medulla

**Figure 2:**
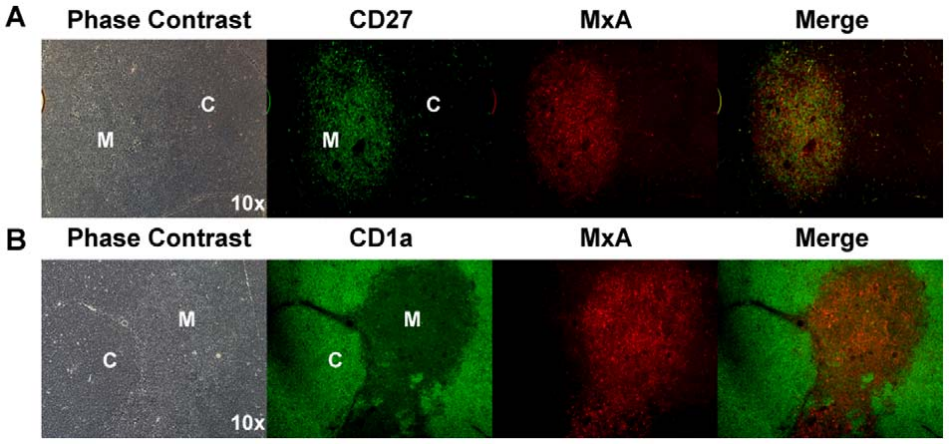
MxA is expressed in the thymic medulla. Postnatal thymus tissue was formalin fixed and paraffin embedded. 4 um sections were mounted on slides. *A*, Thymus tissue was stained with CD1a and MxA followed by Goat anti-mouse IgG1 Alexa Fluor 488 and Goat anti-mouse IgG2a Alexa Fluor 594. *B*, Thymus tissue was stained with antibodies directed against CD27 and MxA followed by Goat ant-mouse IgG1 Alexa Fluor 488 and Goat anti-mouse IgG2a Alexa Fluor 594.

To determine whether type I IFNs are secreted in specific regions in the thymus *in vivo*, we performed immunofluorescence microscopy using an anti-MxA monoclonal antibody. Fetal, postnatal, and adult thymus tissues were stained with CD1a to distinguish the cortex and CD27 to distinguish the medulla. In 3/3 fetal and 9/9 postnatal thymus samples (age 3 months-30 years) MxA co-localized with the CD27^+^ thymocytes in the medulla (Fig.2A), but not with the CD1a^+^ thymocytes in the cortex (Fig. 2B). Within the medulla, both CD27^+^ and CD27^−^ cells expressed MxA (Fig. 2A). To confirm these results, post-natal thymus tissue sections were also stained for expression of IRF-7, a factor required for the transcription of downstream interferon stimulated genes including MxA [2]. Like MxA, IRF-7 was preferentially expressed in the medulla, but not in the cortex (data not shown). Localization of MxA and IRF-7 in areas of the thymus that stain with CD27 confirms that IFN-α/β is secreted within the thymic medulla.

### MxA, IRF-7, and phosphorylated STAT1 are preferentially expressed in mature thymocytes

**Figure 3:**
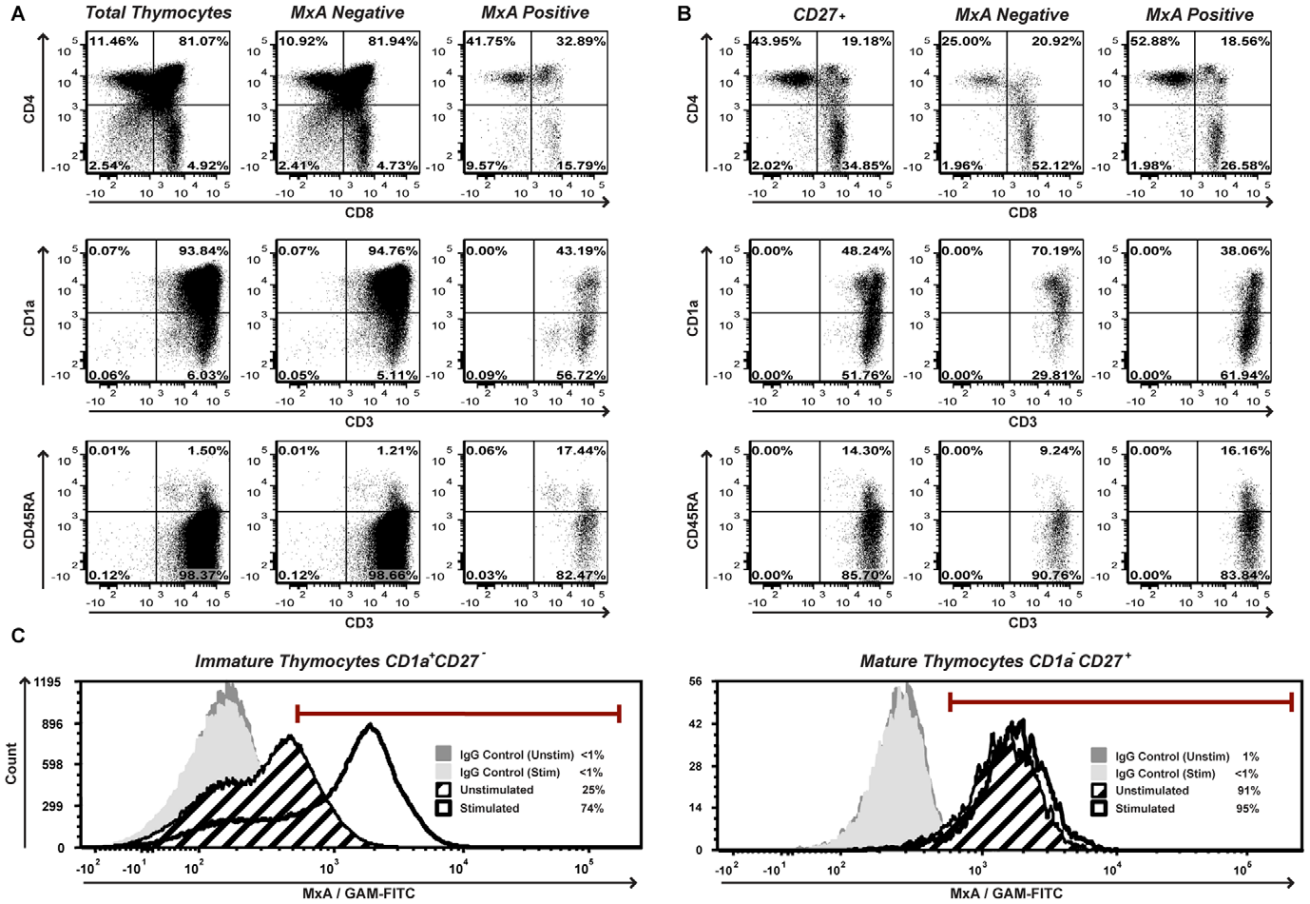
MxA is preferentially expressed in mature thymocytes and exogenous IFN-α only upregulates MxA in immature CD1a^+^CD27^−^ thymocytes. *A, B,* Total thymocytes were stained for surface expression of CD4, CD8, CD1a, CD3, CD27, CD45RA and CD123 combined with intracellular MxA expression. *A,* Differences of thymocyte subset expression patterns in all thymocytes, MxA negative and positive cells are shown. *B*, Differences of thymocyte subset expression patterns in CD27^+^, MxA negative and positive cells are shown. *C,* Total thymocytes were cultured in the presence or absence of 1000 U/mL IFN-α overnight. Cells were stained for surface expression of CD4, CD8, CD1a, CD3, CD27, CD45RA and CD123 combined with intracellular MxA expression. MxA expression in immature and mature thymocyte subsets was determined by gating on CD1a^+^CD27^−^ and CD1a^−^CD27^+^ cells respectively.

We and others have shown that most thymocyte subsets express the receptor for IFN-α (CD118) and can respond to stimulation with type I IFNs [17] ^,^ [34]. If IFN-α is secreted throughout the thymic cortex and medulla then the phenotype of MxA expressing cells should match the phenotypic distribution of all developing thymocytes. To determine the immunophenotypic profile of MxA expressing cells, total thymocytes were stained for cell surface expression of CD4, CD8, CD1a, CD3, CD27, CD45RA and CD123 in combination with intracellular MxA expression. We observed that MxA is preferentially expressed in cells that exhibit a more mature phenotype (ie. CD45RA^+^, CD4^+^ or CD8^+^, CD3^+/hi^, CD1a- and CD27^+^) (Fig. 3A). Furthermore, when focusing on the mature, medullary, thymocyte subsets (ie. CD27^+^ cells), MxA expression was enriched in the mature CD45RA^+^CD4^+^ and CD45RA^+^CD8^+^ cells that are ready to emigrate from the thymus to the periphery (Fig. 3B). These data are consistent with the notion that type I IFNs are secreted locally in the medulla.

We also assessed expression of phosphorylated STAT1 (pSTAT1) and IRF-7, which are upstream of MxA, and dependent on signals received from the type I IFN receptor [2]. Phosphorylated STAT1 (Fig. S1A) and IRF-7 were also preferentially expressed in mature thymocytes (Fig. S1B) confirming the expression pattern observed with MxA. Taken together, the presence of MxA, pSTAT1 and IRF-7 support the notion that type I IFNs are constitutively secreted in the thymus. Furthermore, the phenotype of the cells that have responded to type I IFNs and as a result phosphorylate STAT1 and express MxA and IRF-7 *ex vivo* suggest that type I IFNs are secreted locally within the medulla where mature thymocytes are located.

### Stimulation of thymocytes with exogenous IFN-α upregulates MxA and pSTAT1

While all thymocyte subsets express CD118, we cannot exclude the possibility that immature thymocytes may not respond to stimulation by IFN-α. To test this notion, thymocytes were stimulated with 1000 U/mL recombinant IFN-α overnight and stained for surface expression of CD4, CD8, CD1a, CD3, CD27, CD45RA and CD123 combined with intracellular MxA expression. Stimulation with exogenous IFN-α showed a robust increase in total MxA expression in CD27^−^CD1a^+^ thymocytes, confirming the ability of immature thymocytes to respond to IFN-α. Notably, no further increase in MxA expression in CD27^+^CD1a^−^ mature thymocytes was observed (Fig. 3C). Similarly, pSTAT1 levels increased in immature, but not in mature thymocytes after stimulation with 1000 U/mL exogenous IFN-α (data not shown). These results indicate that all thymocytes have the potential to respond to IFN-α *ex vivo*. However *in vivo,* immature thymocytes are less stimulated and mature thymocytes may have already been stimulated to maximal levels by constitutively produced IFN-α.

### pDC express IFN-α and higher levels of MxA in the thymus compared to other thymocyte subsets

We have previously shown that thymic pDC are necessary for the expression of MxA in response to HIV-1 infection of the thymus [17]. Their role as the natural interferon producing cells suggests that thymic pDC are likely involved in the constitutive secretion of IFN-α and subsequent expression of MxA by thymocytes. Using real-time PCR analysis, we observed that IFN-α mRNA is transcribed in the thymus and that IFN-α transcripts are preferentially transcribed in MACS enriched or sorted pDC compared to total thymocytes or non-pDC (Fig. S2).

**Figure 4:**
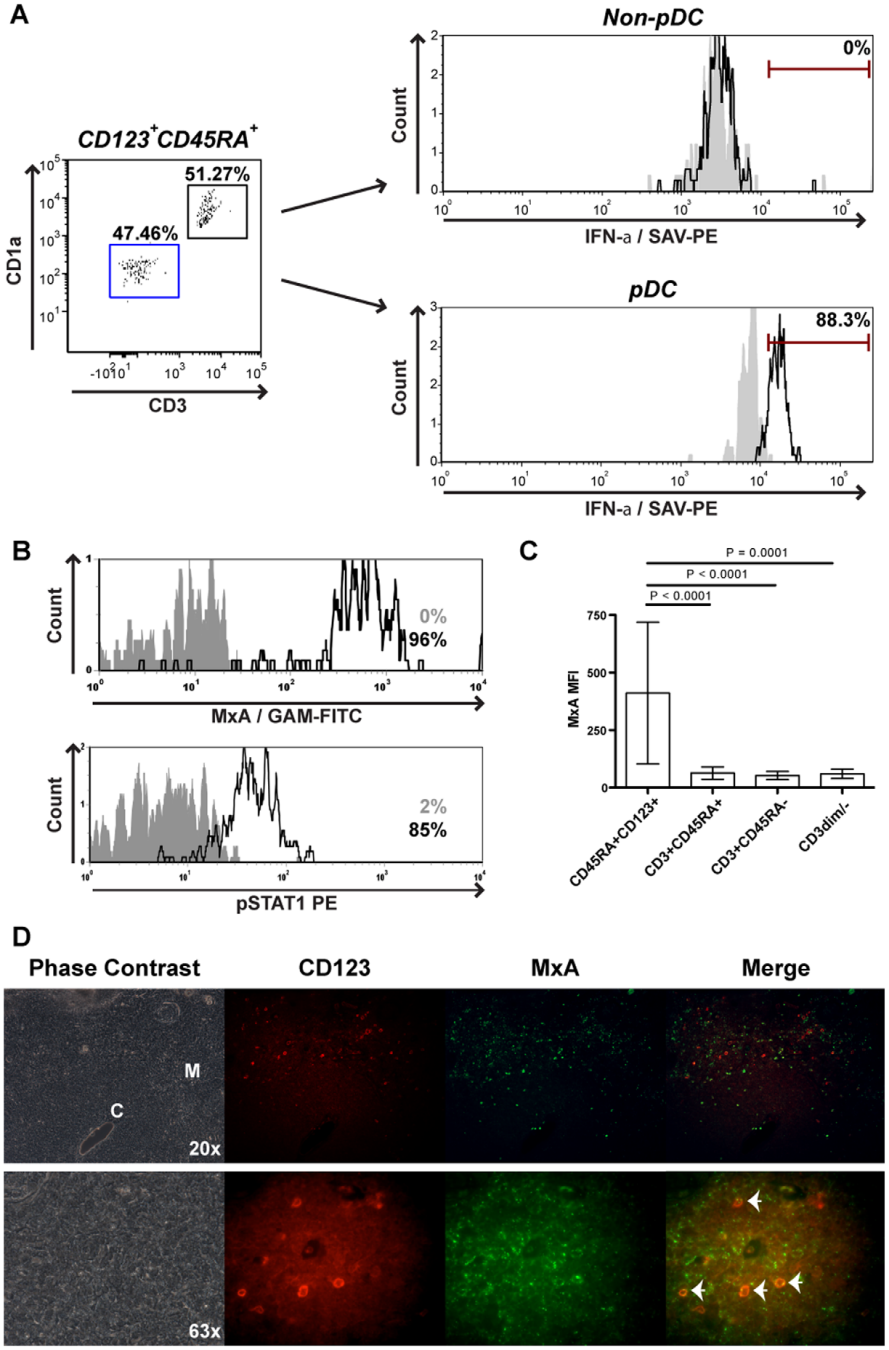
Thymic pDC constitutively express IFN-α, MxA and pSTAT1. *A*, pDC from post-natal thymus express IFN-α. Total thymocytes were stained for surface expression of CD123, CD45RA, CD4, CD8, CD27, CD1a and CD3 and intracellular expression of IFN-α. IFN-α expression of pDC was determined by gating on CD45RA^+^CD123^+/hi^ cells, that lacked CD3 and CD1a. *B*, pDC from post-natal thymus express MxA and pSTAT1. Total thymocytes were stained for surface expression of CD45RA, CD123, and CD3 and intracellular expression of MxA or pSTAT1. MxA and pSTAT1 expression in pDC was determined by gating on CD45RA^+^CD123^+/hi^ cells that lacked CD3. *C*, pDC express the highest level of MxA. Total thymocytes were stained for surface expression of CD45RA, CD123, and CD3 and intracellular expression of MxA. Mean fluorescence intensity (MFI) of MxA expression in pDC was determined by gating on CD3^−^CD45RA^+^CD123^+/hi^ cells. MxA MFI expression in mature and immature thymocyte subsets was determined by gating on CD45RA^+^CD3^+/hi^, CD45RA^−^CD3^+^ and CD3^low/−^ cells. *D,* pDC are located in the medulla of normal thymus. Thymus tissue was formalin fixed and paraffin embedded. 4 um sections were mounted on slides. Thymus tissue was stained for CD123 followed by Rabbit anti-mouse IgG1 and Goat anti-Rabbit Alexa Fluor 594. MxA was conjugated with zenon anti-IgG2a Alexa 488.

To confirm IFN-α protein expression in pDC, we electronically gated on CD123^+^CD45RA^+^ thymocytes which lacked expression of CD1a and CD3, the phenotype of pDC. As can be seen in Figure 4A, >88% of pDC expressed IFN-α, while non-pDC did not. Since it is known that pDC themselves are able to respond to type I IFNs, it is expected that pDC express MxA. By flow cytometry we measured MxA and pSTAT1 expression *ex vivo* in pDC (identified here as CD45RA^+^CD123^+/hi^ cells), isolated from post-natal thymus tissues and found that (89±9%) of pDC are MxA positive (n = 15) (Fig. 4B). In addition,MxA mRNA was also measured and observed to be expressed at high levels in pDC (Fig. S2). Post-natal thymic pDC also expressed pSTAT1 (Fig. 4B) and IRF-7 (data not shown). Furthermore, thymic pDC showed a significantly higher MxA mean fluorescence intensity (3–13 fold, p<0.00001, (n = 15)) than other MxA positive thymocyte subsets (Fig. 4C).

To confirm our observation that pDC express high levels of MxA in the thymus, but not in other lymphoid tissues, we stained single cell suspensions from fetal tissues or adult PBMC for surface expression of CD123, CD45RA, CD3 and intracellular expression of MxA. MxA expression in pDC was determined by gating on CD45RA^+^CD123^+/hi^ cells that lacked CD3. We observed that in 3/3 experiments pDC from the thymus were MxA positive, while pDC from autologous fetal spleen and lymph node lacked MxA expression (Fig. S3). Adult peripheral blood pDC also lacked MxA expression (Fig. S3) and a lack of MxA expression was also observed in Cord blood (data not shown).

To verify MxA expression in thymic pDC and to analyze co-localization with MxA positive thymocytes, post-natal thymus tissue was stained with CD123 and anti-MxA antibodies. In line with previous findings, pDC (CD123^+^) are located within the medulla and at the cortico-medullary junction, but not found within the cortex (Fig. 4D) [18], [19], [35]. As expected from the flow cytometric data, the majority of cells that express high levels of CD123 also expressed MxA (Fig. 4D, Arrows). Moreover, pDC were located in close proximity to thymocytes that expressed MxA (Fig. 4D, Merge). The co-localization of pDC with MxA positive cells in the medulla suggests that pDC are directly responsible for the secretion of IFN-α in the thymus. Furthermore, the finding that MxA is highly expressed in thymic pDC adds value to the notion that type I IFN can act in an autocrine or paracrine manner as a pDC survival factor and is in agreement with a previous report [36].

### The anti-microbial peptide LL-37 co-localizes with pDC in the medulla

**Figure 5:**
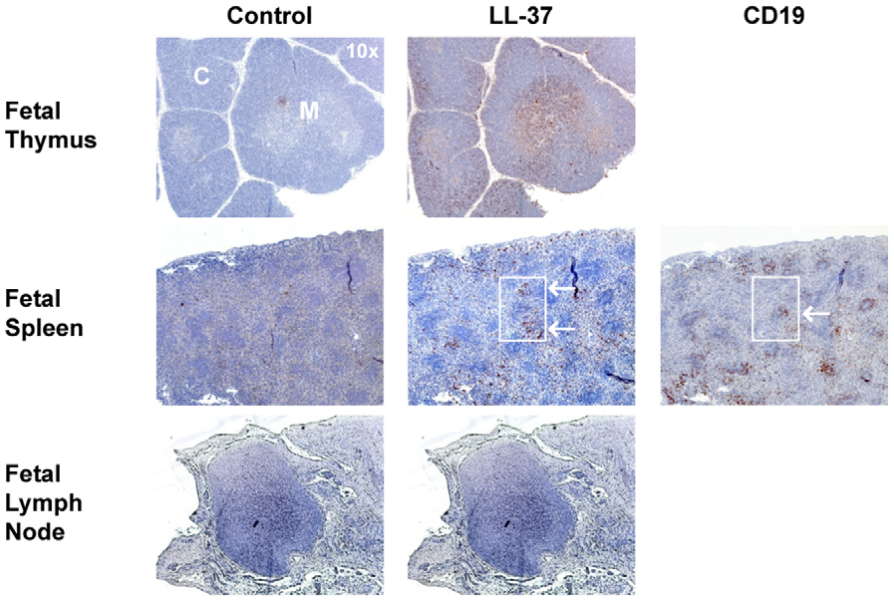
LL-37 is located in the cortex and medulla of the thymus and in non-B cell zones of the fetal spleen. Fetal thymus, spleen, and lymph nodes were formalin fixed and paraffin embedded. 4 um sections were mounted on slides and stained with a monoclonal antibody to LL-37 or CD19 followed by biotinylated rabbit anti-mouse antibody and streptavidin-biotin-peroxidase complex. IgG2a was used as control antibody. (C) cortex; (M) medulla. The fetal tissues were obtained from the same donor.

Our data suggest that there is a thymus-specific trigger for pDC to induce IFN-α/β secretion. Recent publications demonstrated that the anti-microbial peptide LL-37 isolated from psoriasis skin lesions can bind eukaryotic DNA and RNA and trigger peripheral blood pDC to secrete IFN-α in a TLR-9 dependent manner [29], [30]. We hypothesized that expression of LL-37 in the thymus could result in interferon secretion, since DNA/RNA would be readily available from medullary thymocytes undergoing apoptosis as a result of negative selection. To assess whether and where LL-37 protein is expressed, thymic tissue sections were stained with an anti- LL-37 monoclonal antibody. We found that LL-37 was mainly expressed in the medulla in the fetal thymus, although some expression was also observed in the cortex by immunofluorescence (data not shown) and immunohistochemistry (Fig. 5, brown staining). Thus the combined presence of pDC, LL-37, and autologous DNA/RNA derived from negatively selected thymocytes in the medulla can explain the presence of MxA in normal thymus tissue. While LL-37 was not detected in the fetal lymph node (same donor as fetal thymus), it was found in several (n = 3) non-B (CD19^+^) cell zones of the fetal spleen. We speculate that there is likely no/little apoptosis occurring in the fetal spleen resulting in no/limited amounts of autologous DNA/RNA. This could account for the observed lack of MxA expression in lymphocytes and pDC in the fetal spleen (Fig. 1 and Fig. S3).

### Exogenous LL-37 complexed with DNA/RNA upregulates IFN-α

**Figure 6:**
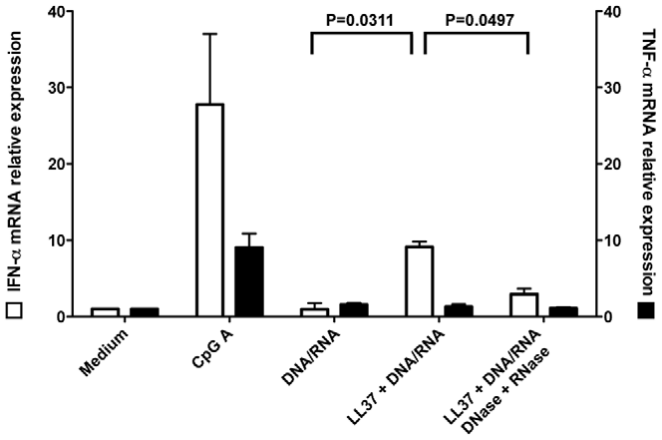
LL-37 upregulates the expression of IFN-α, but not TNF-α in thymic pDC. Sorted pDC were incubated with LL-37 plus or minus eukaryotic DNA/RNA or LL-37-DNA/RNA complex pretreated with DNAse and RNAse. CpG-A 2216 was used as a positive control. Q-PCR was performed for IFN-α and TNF-α mRNA.

Based on findings that IFN-α production by peripheral pDC can be induced by LL-37 complexed with eukaryotic DNA or RNA [29], [30], we examined whether exogenous LL-37 complexed with eukaryotic DNA/RNA increases IFN-α secretion by thymic pDC. PDC were sorted from thymus and stimulated with LL-37 complexed with DNA/RNA obtained from supernatants of apoptotic cells. After 6 hours of culture, we detected 9.1-fold more IFN-α transcripts compared to either medium or eukaryotic DNA/RNA alone (Fig. 6). Notably, pretreatment of the supernatant containing eukaryotic DNA/RNA with DNAse and RNAse partially inhibited IFN-α mRNA expression. Stimulation with the TLR9 agonist CpG-A (2216), which was used as a positive control, induced 3-fold higher levels of IFN-α expression compared to LL37/eukaryotic RNA/DNA complexes. In addition, we found that autologous DNA from apoptotic thymocytes complexed with LL-37 induced MxA mRNA expression and IFN-α production in the supernatant of cultured pDC (data not shown). Collectively, these results provide evidence for the notion that LL-37 when complexed with DNA/RNA is able to increase IFN-α production, which may subsequently induce MxA expression in the medulla of the thymus. Notably, while stimulation of pDC with CpG-A also resulted in the production of TNF-α, stimulation with LL-37 complexed with DNA/RNA did not (Fig. 6). This result suggests that thymic pDC do not promote a pro-inflammatory state in the thymic medulla.

## Discussion

Here we show that the interferon induced protein MxA is constitutively expressed in post-natal and fetal thymus, but not in peripheral lymphoid tissues including fetal spleen and lymph nodes (from the same donor as the thymus). In addition, we show that the majority of thymic pDC express high levels of IFN-α. This together with their medullar localization strongly suggests that thymic pDC are responsible for inducing MxA expression exclusively in the medulla. Finally, we observed that the anti-microbial peptide LL-37 is expressed in the thymus, and that this in complex with eukaryotic DNA/RNA can stimulate thymic pDC to express IFN-α *in vitro*. Collectively, our findings imply that type I IFNs, which are constitutively expressed in the medulla, may have a direct role on the development of T cells in the thymus.

MxA has been used as a reliable marker to detect type I IFN in clinical settings, including SLE and hepatitis C infection [37], [38], as it is exclusively expressed after engagement of type I IFNs to its receptor [9]. Hence, we conclude that type I IFNs are constitutively expressed in the thymus, but not in the spleen or lymph node. This is consistent with our previous observations that IFN-α expressing pDC are present in the human fetal thymus/liver implant in the SCID-hu mouse [17]. Our conclusion is further enforced by the findings that pSTAT1 and IRF-7, which are both activated upon type I IFN signaling, are expressed in the normal thymus [33]. The fact that not only post-natal but also fetal thymus tissue harbors a type I IFN stimulated gene signature endorses the notion that this phenomenon occurs in the absence of viral or bacterial infection and is further supported by the presence of “immunoreactive” IFN-α in fetal thymus tissues described many years ago [39].

A notable finding in our study is that MxA expressing thymocytes (Fig.1) and pDC (Fig. S3), were only present in fetal and postnatal thymus tissues, but not in fetal spleen or lymph node or in adult peripheral blood. This prompted us to search for a stimulus which was only present in the thymus, but not in the peripheral lymphoid organs. We considered the antimicrobial peptide LL-37 as a potential candidate based on a recent report that LL-37 isolated from psoriasis skin lesions can bind DNA and trigger peripheral blood pDC to secrete IFN-α via TLR-9 [29]. The same group reported that eukaryotic RNA/LL-37 complexes trigger the secretion of IFN-α by pDC through TLR7 activation [30]. More recently it was demonstrated that IFN-α secretion in response to CpG-ODNs can also be mediated by the cytosolic sensors aspartate-glutamate-any amino acid-aspartate/histidine (DExD/H)-box helicase 36 (DHX36) and DHX9, which is TLR7 and 9 independent but MyD88 dependent [40]. CAMP1, the gene encoding LL-37, is expressed in many tissues with the highest expression levels in bone marrow and thymus [31]. Our results show that LL-37 is mainly expressed in the thymic medulla, although some expression was also observed in the cortex. The co-localization of pDC and LL-37 in the thymic medulla where nucleic acids may be present from thymocytes undergoing apoptosis as a result of negative selection [32], suggests that LL-37 is a likely trigger for the constitutive secretion of IFN-α in the thymus. Although it is known that macrophages will rapidly phagocytose apoptotic cells, we previously showed that apoptotic CD4^+^ thymocytes can be identified in the human thymus [41]. We also found LL-37 expression in the fetal spleen, but as there is little apoptosis in this organ, it can be assumed that the lack of DNA accounts for the lack of IFN-α secretion and subsequent MxA expression in the fetal spleen.

We previously reported that MxA is highly expressed in pDC and mature thymocytes in response to CpG or HIV-1 induced type I IFN production [17]. In the current study we show that pDC constitutively express IFN-α in the absence of microbial stimulation, and are likely the main source of IFN-α secretion in the thymus. The observation that pDC in normal thymus express high levels of MxA indicates that they also responded to secreted IFN-α in an autocrine fashion. This is consistent with the notion that pDC employ type I IFNs for their survival [36]. It is of note, that the mean fluorescence intensity of MxA is significantly higher in pDC than in other thymocyte subsets, although the explanation for this is currently unclear. It is possible that this can be attributed to increased responsiveness to type I IFNs due to higher expression levels of the type I IFN receptor or downstream signaling molecules. Alternatively, it can be envisioned that negative regulators of the JAK/STAT pathway, such as SOCS proteins, are differentially activated in pDC compared to thymocytes.

Our current data using immunofluorescence, and those of Meager *et al* [42], indicate that MxA expressing cells are mainly present in the thymic medulla. We confirmed these findings by flow cytometry as detailed characterization of MxA^+^ cells enriches for cells with a medullary phenotype, including CD3^+/hi^CD27^+^CD1a^−^CD45RA^+^. Meager *et al.* [42], in a study of anti-interferon autoantibodies in autoimmunity, showed that resident macrophages expressed IFN-α in the postnatal thymus, but did not express MxA and are therefore likely only storing IFN-α and not actively secreting it. The data presented here suggest that IFN-α is continuously produced in the thymic medulla by pDC resulting in MxA expression in medullary thymocytes and pDC. However it is still unclear what role this cytokine plays in thymopoiesis *in vivo*. We previously reported that IFN-α is a reversible inhibitor of human T cell development [16]. Murine studies found that exogenous IFN-α interfered with early T and B cell development at stages where IL-7 stimulation is necessary [13]. Utilizing the OP-9 culture system to mimic thymopoiesis *in vitro*, we showed that IFN-α inhibits early stages of thymocyte development as it delayed the acquisition of CD1a on developing CD34^+^ thymic progenitor cells [16]. In addition we found that IFN-α inhibited later stages of T cell development from CD3^+hi^CD1a^+/hi^ to CD3^+/hi^CD1a^-^ [16]. Cells of these developmental stages, which are present at the cortico-medullary junction, coincide with stages where thymocytes are responsive to IL-7 induced proliferation. Taken together, these data suggest that IFN-α may serve to control the rate of thymopoiesis. Another attractive consideration is that IFN-α secretion may play a role in negative selection. Autoreactive T cells are negatively selected in the medulla and are removed via apoptosis (reviewed in[43]) induced by several mechanisms including TRAIL-mediated cell death (reviewed in[44]). This is consistent with reports demonstrating that the expression of MxA increases a cell's susceptibility to cell death [45], [46]. Hence, it is possible that mature MxA^+^ thymocytes may be more sensitive to cell death as a result of MxA expression. The increased susceptibility to cell death may reduce the signal intensity necessary to delete autoreactive T cells. The expression of MxA in mature thymocytes may aid in reducing the threshold needed for negative selection.

Finally, we believe that our data have implications in the pathogenesis of CCR5-tropic compared to CXCR4-tropic HIV-1 infection in the thymus. Previously we reported that the proportion of pDC expressing IFN-α increased after infection with either CXCR4 or CCR5-tropic HIV-1 [17]. We and others have shown that infection with CCR5-tropic viruses leads to infection of mature thymocytes within the thymic medulla [47], [48], [49], [50]. We speculate that the constitutive expression of ISG in the medulla may make this region relatively more resistant to infection. This notion is in line with the reduced pathogenicity of CCR5-tropic strains when compared to CXCR4-tropic strains [50], [51]. In contrast, CXCR4-tropic strains may be more pathogenic due to the fact that they initially infect cells located within the cortex where we did not observe constitutive ISG expression [47], [49], [51], [52]. It is tempting to speculate that by infecting cells that are distantly located from pDC and the ISG, these virus strains are able to replicate much more efficiently. This suggests that constitutive IFN-α secretion, in addition to its potential role in T cell development, is essential in inhibiting acute infection with CCR5-tropic HIV-1.

## Supporting Information

Figure S1 **pSTAT1 and IRF-7 are preferentially expressed in mature thymocytes.** Total thymocytes were stained for surface expression of CD4, CD8, CD1a, CD3 and CD27 and intracellular expression of pSTAT1 (A) or IRF-7 (B). Differences of thymocyte subset expression patterns in all thymocytes and pSTAT1 and IRF-7 negative and positive cells are shown.
(TIF)

Figure S2 **Thymic pDC constitutively express high levels of IFN-α mRNA.** mRNA was prepared from postnatal total thymocytes, BCDA4-MACS enriched pDC and the BDCA4 negative fraction, and from sorted pDC. Q-PCR was performed for IFN-α mRNA.
(TIF)

Figure S3 **pDC from post-natal and fetal thymus express MxA, but not pDC from fetal spleen or fetal lymph node or adult PBMC.** Cells from fetal tissues or adult PBMC were stained for surface expression of CD123, CD3 and CD45RA and intracellular expression of MxA. MxA expression in pDC was determined by gating on CD45RA^+^CD123^+/hi^ cells.
(TIF)
