# Supplementary figures and images for: IFN-α Is Constitutively Expressed in the Human Thymus, but Not in Peripheral Lymphoid Organs
Source: PLoS One. 2011 Aug 31;6(8):e24252. doi: 10.1371/journal.pone.0024252 (PMC3164161; doi:10.1371/journal.pone.0024252)

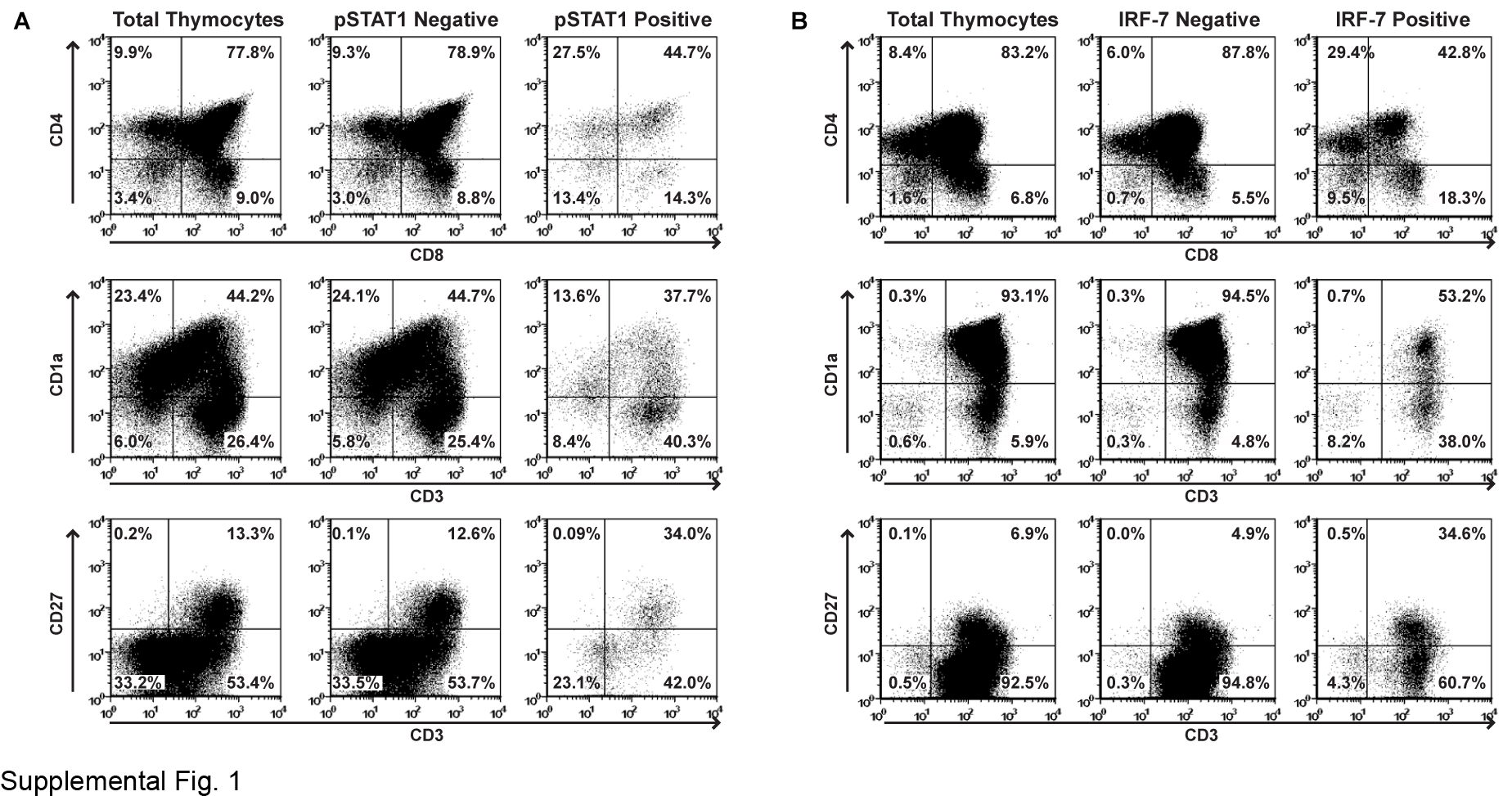

Supplement: Figure S1 — pSTAT1 and IRF-7 are preferentially expressed in mature thymocytes. Total thymocytes were stained for surface expression of CD4, CD8, CD1a, CD3 and CD27 and intracellular expression of pSTAT1 (A) or IRF-7 (B). Differences of thymocyte subset expression patterns in all thymocytes and pSTAT1 and IRF-7 negative and positive cells are shown. (TIF) [file pone.0024252.s001.tif]

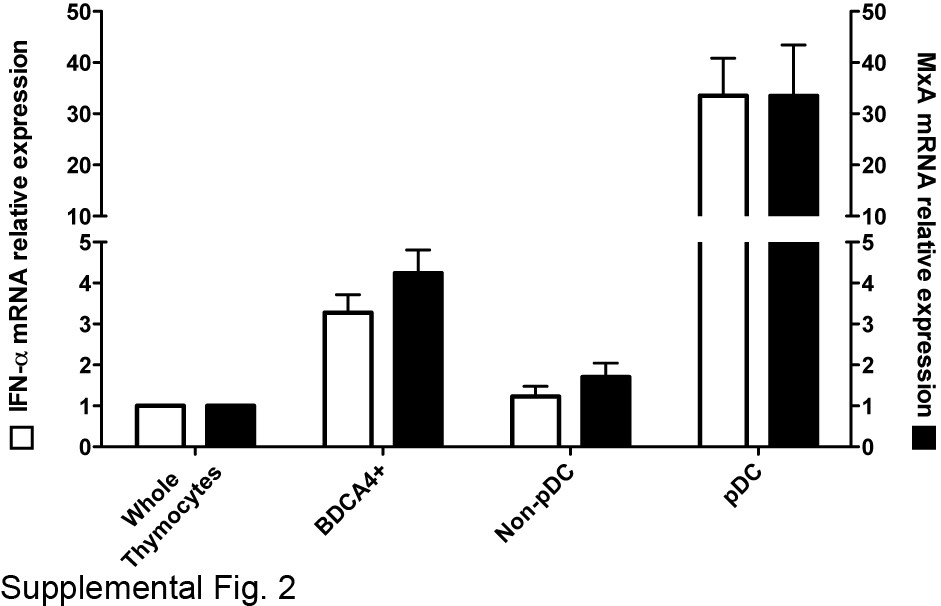

Supplement: Figure S2 — Thymic pDC constitutively express high levels of IFN-α mRNA. mRNA was prepared from postnatal total thymocytes, BCDA4-MACS enriched pDC and the BDCA4 negative fraction, and from sorted pDC. Q-PCR was performed for IFN-α mRNA. (TIF) [file pone.0024252.s002.tif]

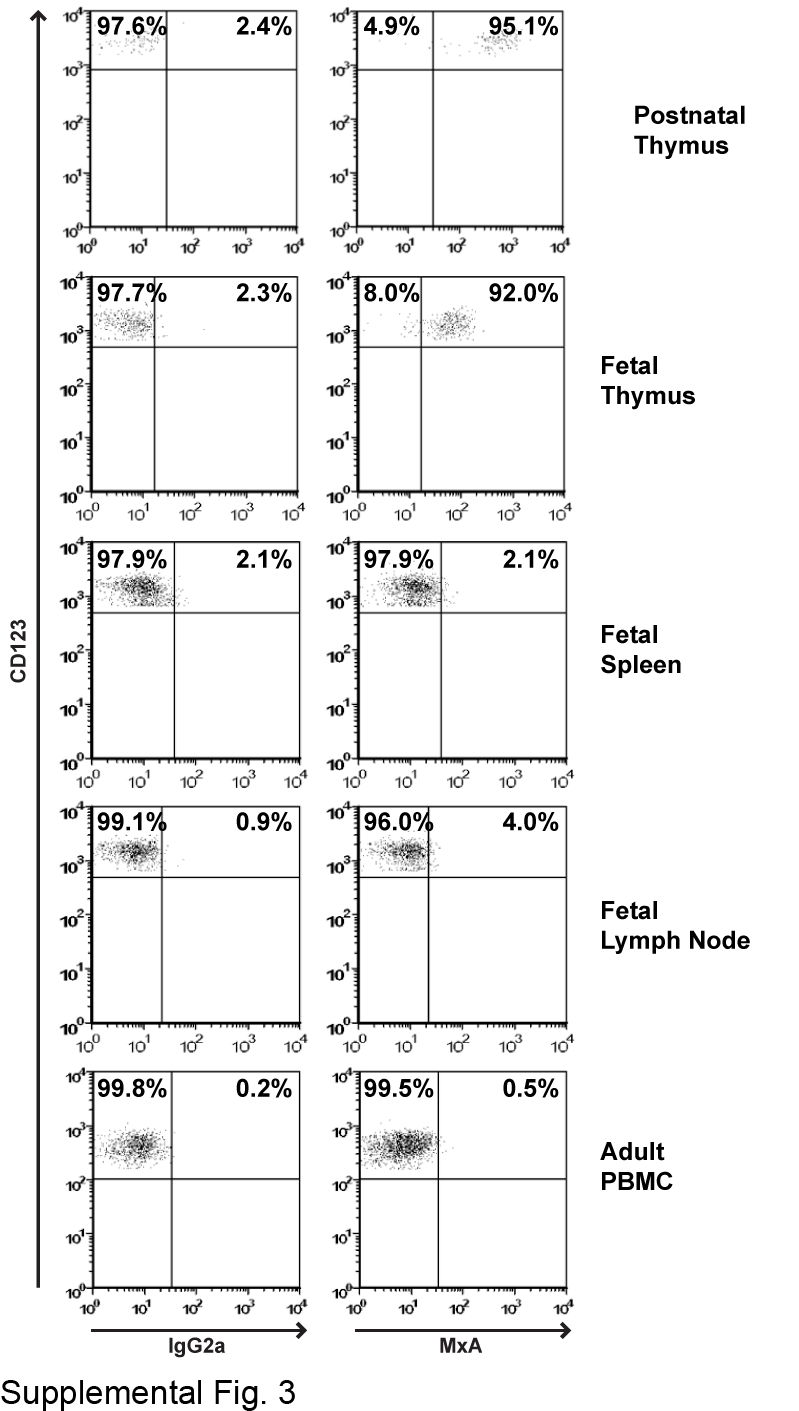

Supplement: Figure S3 — pDC from post-natal and fetal thymus express MxA, but not pDC from fetal spleen or fetal lymph node or adult PBMC. Cells from fetal tissues or adult PBMC were stained for surface expression of CD123, CD3 and CD45RA and intracellular expression of MxA. MxA expression in pDC was determined by gating on CD45RA+CD123+/hi cells. (TIF) [file pone.0024252.s003.tif]
